# 

**Published:** 1894-09

**Authors:** 


### CONTENTS.

ORIGINAL ARTICLES—

The Cause of the Increase of Diseases of Women, and Their Prevention. By Geo. W. Christian, M.D., Houston, Tex........ ...... ........ 466

International Congress of Hygiene and Demography. By W. H. Dalrymple, M.R.C.V.S. (Eng.)........................................ 470

A Case of Imperforate Hymen. By Andrew Boyd, M.D., Scottsboro, Ala.............................................. .. 473

Spontaneous Cure of a Nævus Maternus—Large Vascular Tumor Occupying Side of Neck. By A. Bethune Patterson, M.D., Atlanta, Ga.................................................. 477

A Case of Abdominal Hysterectomy in which the Ureter was Resected and Implanted into the Bladder. By Charles B. Penrose, M.D., Philadelphia, Pa............................................. 479

Osteosarcoma Treated with Pyoktanin. Hy J. I. Darby, M.D., Americus, Ga.................................................. 481

SOCIETY REPORTS—

Chattanooga Medical Society.............................................. 483

BOOK REVIEWS—

Lectures on Auto-Intoxication in Disease, or Self-Poisoning of the Individual. By Charles Bouchard.......................................... 487

In Press—A New and Concise Text-Book on Hygiene. By Mary Taylor Bissell, New York.............................................. 488

SELECTIONS AND ABSTRACTS—
Summer Complaint. By Paul J. Barcus, M.D., Crawfordsville, Ind... 489
Sulphuric-Acid Paste in the Treatment of Epithelioma of the Face. By E. Oliver Belt, M.D., Washington, D. C ............ ....................495
Chancroid of Eight Years' Standing. By R. M. Kirley, M.D., St. Louis.................. ............ ........... ........ .............................. 497
Chronic Pelvic Peritonitis........ ........ ........... ..................... .... ........ 498
Phagedenic Chancroids ............................. ................................. 498
Some Remarks Concerning Mercuric Bromide of Gold and Arsenic, with Especial Reference to Its Use in the Treatment of Neurotic Conditions of Specific Origin........ ...................... ..... ............... 500
The Therapeutics of Sexual Weakness ........................................ 504
Neebe's Treatment of Sweating Feet by Crude Hydrochloric Acid...... 507

EDITORIAL—
The Tri-State Medical Society of Alabama, Georgia, and Tennessee.....508
The American Academy of Medicine........................................ 510

EDITORIAL NOTES...................................................... ............ 512
PRESCRIPTION DEPARTMENT............................... ...............514, 515
Nasal Catarrh—To Harden the Skin of Bedridden Patients in Order to Prevent Bedsores—Gonorrhea—For Epilepsy—Antiseptic Powder—For Amenorrhea—Pertussis (Whooping Cough)—Treatment of Gastro Internal Catarrh in Children.
SPECIAL NOTES..... ..................................................... 516, 518, 520, 522

